# Equity and efficiency of health resource allocation in township health centers in Sichuan Province, China

**DOI:** 10.1371/journal.pone.0299988

**Published:** 2024-03-05

**Authors:** Minghua Zhou

**Affiliations:** Department of Administration Office, Luzhou People’s Hospital, Luzhou, Sichuan, China; New York University Grossman School of Medicine, UNITED STATES

## Abstract

**Objective:**

To analyze the equity and efficiency of health resource allocation in township health centers in Sichuan Province, and to provide a scientific basis for promoting the development of township health centers in Sichuan Province, China.

**Methods:**

The Lorenz curve, Gini coefficient and health resource density index were used to analyze the equity of health resource allocation in township health centers in Sichuan Province from 2017 to 2021, and data envelopment analysis(DEA) was used to analyze the efficiency of health resource allocation in township health centers in Sichuan Province from 2017 to 2021.

**Results:**

The Gini coefficient of health resources of township health centers in Sichuan Province is below 0.2 by population in addition to the number of beds in 2020–2021 and practicing (assistant) physicians in 2021, and the Gini coefficient of health resources of township health centers in Sichuan Province is above 0.6 by geography. The Lorentz curve of health resources of township health centers in Sichuan Province is closer to the equity line by population allocation and further from the equity line by geographical allocation. The average level of township health centers in Sichuan Province is used as the standard to calculate the health resource density standard index(W) of each region, the Ws of Panzhihua, Ganzi, Aba and Liangshan are less than 1, and the Ws of Ziyang, Neijiang, Deyang and Meishan are greater than 1. The overall efficiency of township health centers in Sichuan Province in 2017 and 2021 is 1, and the DEA is relatively effective. The overall efficiency of township health centers in Sichuan Province in 2018 and 2019 is not 1, and the DEA is relatively ineffective. The overall efficiency of all health resources in Mianyang and Ziyang is 1, and the DEA is relatively effective. The overall efficiency of all health resources in Suining, Neijiang, Yibin, Aba and Ganzi is not 1, and the DEA is relatively ineffective.

**Conclusion:**

The equity of health resource allocation by population is better than that by geography in township health centers in Sichuan Province. Combining population and geographical factors, the health resource allocation of Panzhihua, Ganzi, Aba and Liangshan is lower than the average level of Sichuan Province. The efficiency of health resource allocation in township health centers in Sichuan Province is low.

## Introduction

Equity and efficiency are important elements of health resource allocation. Full utilization of health resources is a prerequisite for equitable allocation, and equitable allocation is helpful in improving the efficiency of utilization. It is important to continuously optimize the allocation of health resources and improve the equity and efficiency of health resource allocation to promote the development of health care [1].

The township health center is the foundation of the primary medical and health care service system [2], and performs the functional tasks of treating common diseases, multiple diseases and providing basic public health services for rural residents. Sichuan Province has always attached importance to the development of primary medical and health care, and promoted the construction of standardized township health centers, which gradually increased the number of primary health resources and gradually improved medical conditions. With the development of the economy and the continuous improvement of the basic medical insurance system, the demand for medical services of urban and rural residents has been unleashed, and the problems of insufficient overall medical and health resources and weak capacity of primary medical services in township health centers in Sichuan Province have been highlighted [3], and the supply level of primary medical and health services needs to be further improved [4]. The "Fourteenth Five-Year Plan" for health development in Sichuan Province proposes to strengthen the primary health care service system, build a new structure of primary health care with county-level hospitals as the leader and township health centers and community health service centers as the backbone, so as to alleviate the downward trend in the proportion of primary care visits [5]. In order to improve the overall health level of primary residents during the 14th Five-Year Plan period, and to promote the rational use of medical and health resources by the primary population, it is important to fairly and reasonably allocate medical and health resources in township health centers and improve the medical service capacity of township health centers.

The existing studies on equity and efficiency of health care services in China mainly focus on the equity of health resources allocation in rural three levels [6], the efficiency of medical services in county-level public hospitals [7], the equity and efficiency of health resources in public hospitals [8], and the equity and efficiency of China’s health care service system [9], and few studies have been conducted on the equity and efficiency of township health centers in Sichuan Province [10]. Therefore, this study analyzed the equity of health resource allocation in township health centers in Sichuan Province by using Lorenz curve, Gini coefficient(G) and health resource density index(HRDI), and analyzed the efficiency of health resource allocation in township health centers in Sichuan Province by using data envelopment analysis(DEA), in order to provide a scientific basis for promoting the development of medical and health resources in township health centers in Sichuan Province.

## Methods

### Data sources and statistical analysis

Data on health resources and service utilization of township health centers in Sichuan Province were obtained from the Sichuan Health Statistical Yearbook (2017–2021), and population numbers and geographic areas were obtained from the Sichuan Statistical Yearbook (2018–2022).

### Indicators of equity and efficiency

The number of beds, health technicians, licensed (assistant) physicians, and registered nurses were selected as evaluation indicators for the equity of health resource allocation in township health centers in Sichuan Province. The number of beds, health technicians, licensed (assistant) physicians and registered nurses were selected as input indicators, and the number of consultations and hospital admissions as output indicators to evaluate the efficiency of health resource allocation in township health centers in Sichuan Province.

### Gini coefficients and Lorenz curves

The Lorenz curves are obtained by first ranking each region in ascending order of health resources per capita, then plotting the percentage of cumulative population on the x-axis and the percentage of cumulative health resources on the y-axis. The diagonal line is the absolute equity line, and the degree of curvature of the curve indicates the degree of equity in health resource allocation [11]. The closer the curve is to the diagonal line, the more equity it indicates, and the farther the curve is from the diagonal line, the more inequity it indicates.

The Gini coefficients are a measure of equity calculated from the Lorenz curves [12]. The closer the Gini coefficient is to 0, the better the equity is, and the closer the Gini coefficient is to 1, the worse the equity is. The Gini coefficient below 0.2 means absolute equity, 0.2~0.3 means relatively fair, 0.3~0.4 means basically reasonable [13], 0.5 or above means a wide gap, and 0.6 or above is a dangerous state of high inequality. The formula is:

$$G=2\times [0.5-1÷2\sum_{i=1}^{n}\left(X_{i}-X_{i-1})(Y_{i}+Y_{i-1}\right)]$$

where X_0_ = 0, Y_0_ = 0, n is the number of regions, X_i_ is the cumulative percentage of population (geographic area), and Y_i_ is the cumulative percentage of health resources.

### Health resources density index (HRDI)

The HRDI integrates the influence of population and geographic area factors on the allocation of health resources and is highly practical for sparsely populated regions [14]. The formula is.


$$HRDI=\sqrt{\frac{Health\;resources}{Per\;1,000\;population}\times \frac{Health\;resources}{Per\;square\;kilometer}}$$

Taking the average level of township health centers in Sichuan Province as a standard, the health resource density standard index (W) was used to evaluate the relative level of township health centers in each region, where W is greater than 1 indicates that the allocation of the region is higher than the average level, and W is less than 1 indicates that the allocation of the region is lower than the average level. The formula is:

$$W=\frac{{HRDI}_{region}}{{HRDI}_{standard}}$$

### Data envelopment analysis (DEA)

DEA is an evaluation method that can be used to measure the relative efficiency of decision units with multiple inputs and outputs [15]. The overall efficiency, technical efficiency and scale efficiency of health resource allocation were obtained by DEA, and the overall efficiency of 1 indicates the relative efficiency of DEA [16]. In this study, the DEA-BCC model was used to calculate the relative efficiency with input-oriented and year-based decision units.

### Statistical methods

The database was created in Excel 2010, and Excel 2010 was used to plot the Lorentz curve and calculate the Gini coefficient and the HRDI, and DEAP 2.1 was used for DEA.

### Ethics statement

The data of the Statistical Yearbook are publicly available. Ethical approval is not needed because there is no secondary data for any personal information.

## Results

### The current situation of health resources in township health centers in Sichuan Province in 2021

**Fig 1:**
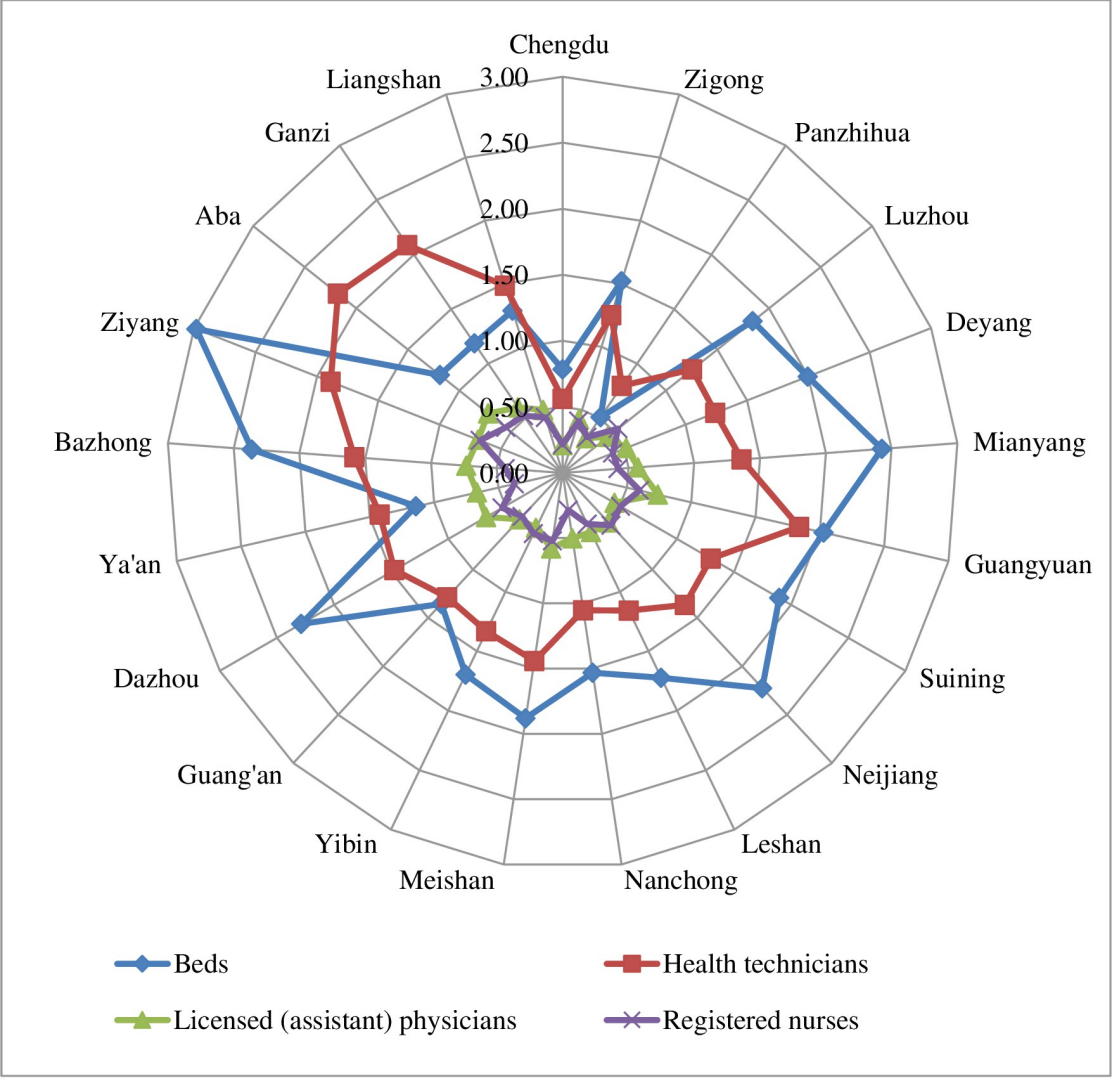
Number of health resources per 1,000 population in township health centers in Sichuan Province in 2021.

In 2021, the allocation of health resources per 1,000 population in township health centers was better in Ziyang and Guangyuan, and worse in Chengdu and Panzhihua (Fig 1).

**Fig 2:**
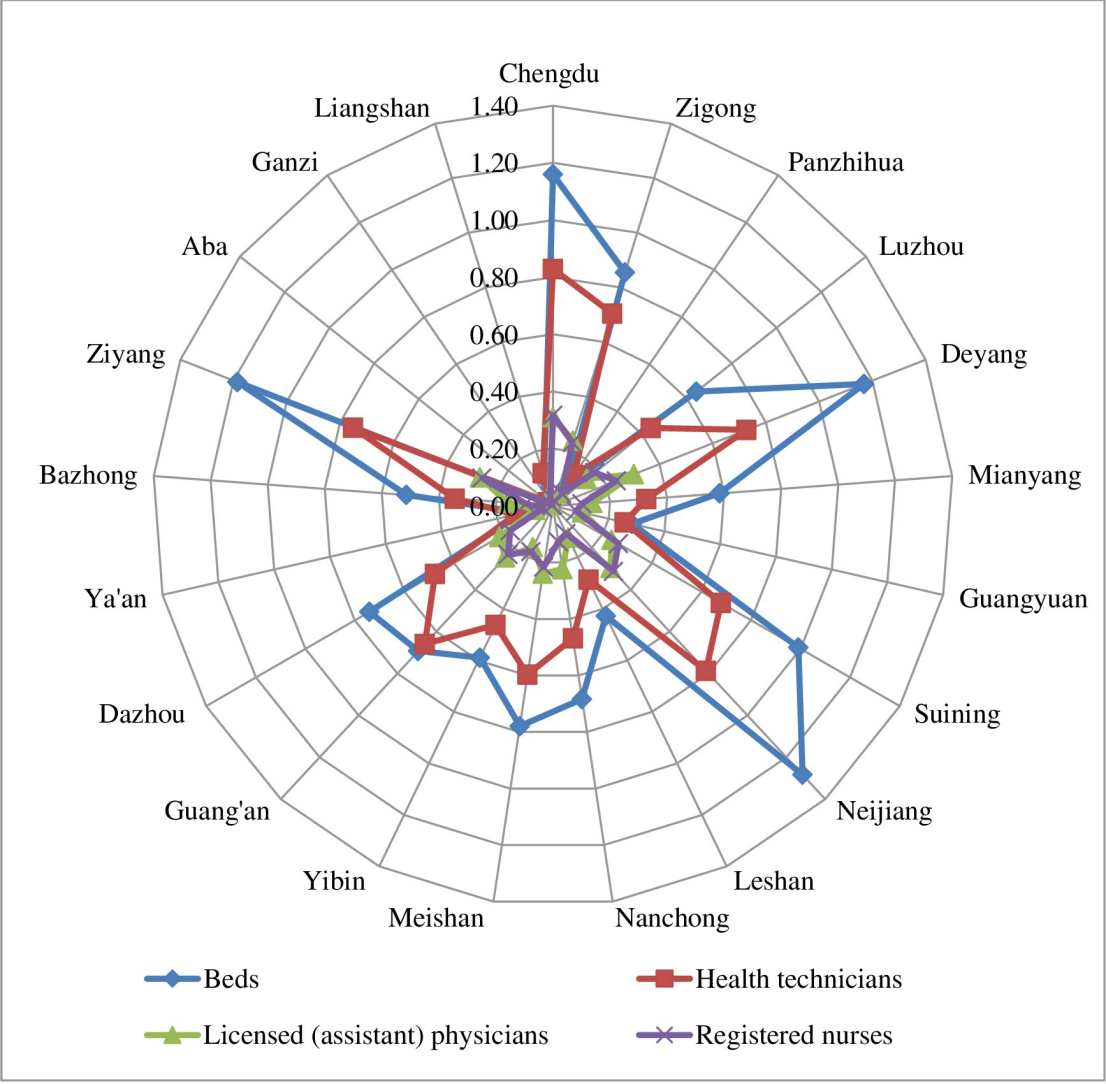
Number of health resources per 1,000 square kilometers in township health centers in Sichuan Province in 2021.

In 2021, the allocation of health resources per square kilometer in township health centers was better in Chengdu, Deyang, Neijiang, and Ziyang, and worse in Ganzi, Aba, and Liangshan (Fig 2).

### Gini coefficient of health resources in township health centers in Sichuan Province

The Gini coefficient of health resources by population allocation in township health centers in Sichuan Province in addition to the number of beds in 2020–2021 and practicing (assistant) physicians in 2021 is below 0.2, indicating that the allocation of health resources is in an absolutely equitable state. The Gini coefficient of health resources allocation by geography allocation in township health centers in Sichuan Province is above 0.6, indicating that the allocation of health resources is in a highly inequitable state. The equity of health resource allocation by population is better than the equity of allocation by geography (Table 1).

**Table 1:** Gini coefficient of health resources in township health centers in Sichuan Province.

Year	By population				By geography			
	Beds	Health technicians	Licensed (assistant) physicians	Registered nurses	Beds	Health technicians	Licensed (assistant) physicians	Registered nurses
2017	0.14	0.13	0.14	0.12	0.66	0.61	0.65	0.64
2018	0.15	0.14	0.13	0.12	0.66	0.61	0.65	0.64
2019	0.16	0.14	0.14	0.13	0.66	0.61	0.64	0.64
2020	0.21	0.18	0.19	0.18	0.66	0.61	0.63	0.64
2021	0.22	0.19	0.20	0.19	0.66	0.61	0.62	0.64

### Lorentz curve of health resources in township health centers in Sichuan Province

**Fig 3:**
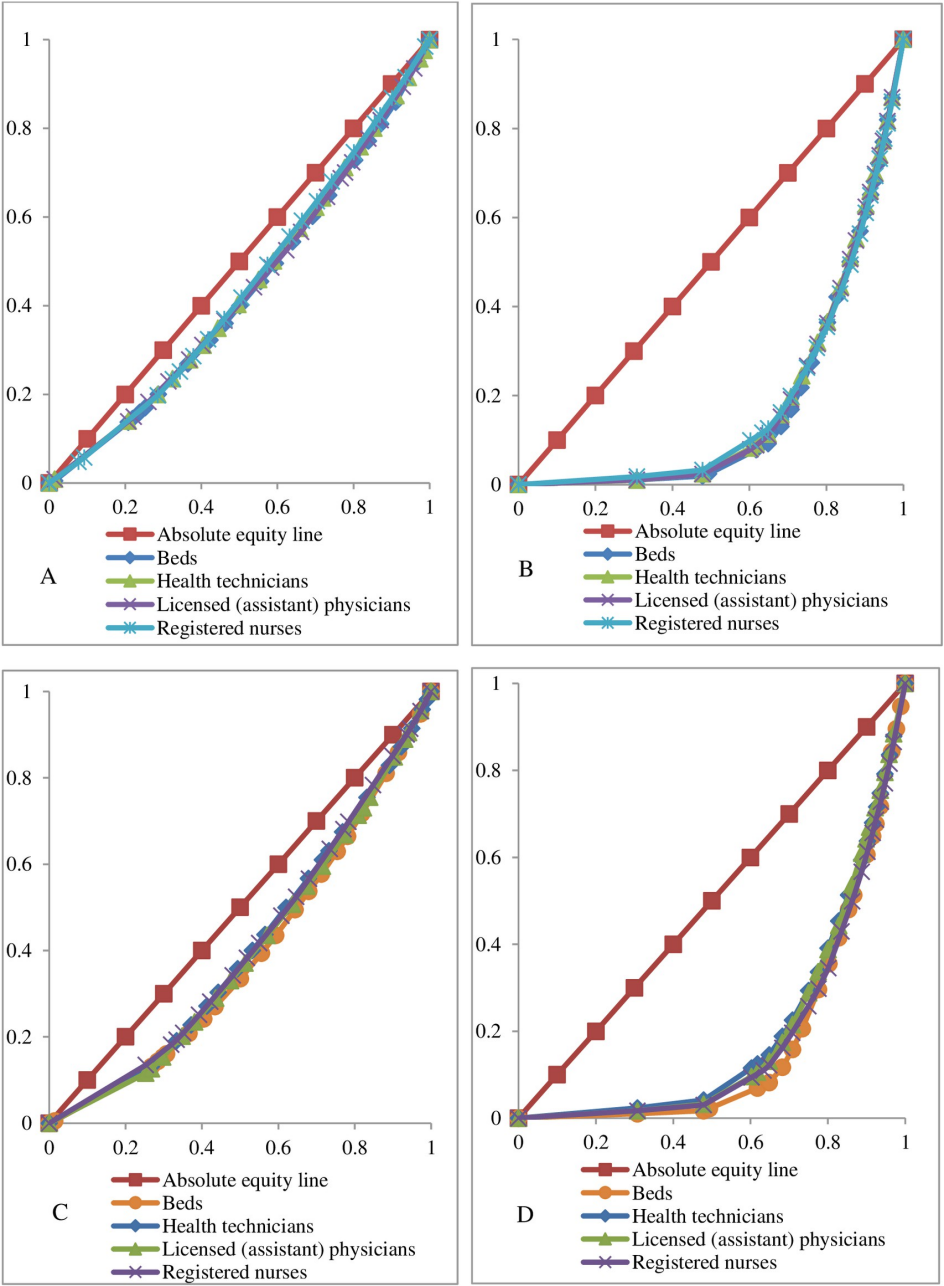
Lorentz curves of health resources in township health centers in Sichuan Province in 2017(A,B) and 2021 (C,D), A and C show Lorentz curves by population allocation, B and D show Lorentz curves by geography allocation.

From Fig 3, the Lorenz curve of health resource allocation by population in township health centers in Sichuan Province is closer to the absolute equity line, and the Lorenz curve of allocation by geography is farther from the absolute equity line, indicating that the equity of health resource allocation by population is better than the equity of allocation by geography.

### The Ws of health resources in township health centers in Sichuan Province

Integrating the factors of population and geographical area, and taking the average level of township health centers in Sichuan Province as the standard, the Ws of each region was calculated as shown in Table 2. The Ws of health resources in Panzhihua, Ganzi, Aba, and Liangshan are less than 1, indicating that the allocation of health resource in these regions is lower than the average level in Sichuan Province. The Ws of health resources in Ya’an are less than 1, except for practicing (assistant) physicians from 2019 to 2021, indicating that the health resource allocation is lower than the average level in Sichuan Province. The Ws of health resources in Ziyang, Neijiang, Deyang and Meishan are greater than 1.8, indicating that the health resource allocation in these regions is much higher than the average level in Sichuan Province.

**Table 2:** The Ws of health resources in township health centers in Sichuan Province.

Region	Beds					Health technicians					Licensed (assistant) physicians					Registered nurses				
	2017	2018	2019	2020	2021	2017	2018	2019	2020	2021	2017	2018	2019	2020	2021	2017	2018	2019	2020	2021
Chengdu	1.75	1.77	1.69	1.50	1.46	1.73	1.70	1.67	1.45	1.40	1.72	1.73	1.66	1.40	1.34	1.86	1.86	1.84	1.59	1.56
Zigong	1.68	1.65	1.66	1.76	1.74	1.86	1.87	1.89	1.88	1.93	1.82	1.80	1.80	1.71	1.67	1.93	1.97	1.93	1.85	1.85
Panzhihua	0.34	0.34	0.34	0.34	0.32	0.60	0.60	0.63	0.67	0.66	0.59	0.59	0.65	0.69	0.67	0.66	0.65	0.69	0.77	0.79
Luzhou	1.58	1.59	1.64	1.66	1.67	1.48	1.53	1.54	1.59	1.52	1.29	1.34	1.35	1.44	1.35	1.91	1.92	1.95	1.98	1.90
Deyang	2.10	2.30	2.40	2.40	2.34	2.09	2.18	2.10	2.07	1.95	2.36	2.43	2.31	2.22	2.09	1.99	2.02	1.97	1.96	1.87
Mianyang	1.84	1.78	1.77	1.77	1.82	1.49	1.44	1.39	1.37	1.37	1.53	1.57	1.51	1.49	1.48	1.30	1.30	1.24	1.20	1.24
Guangyuan	1.18	1.20	1.25	1.30	1.16	1.24	1.32	1.33	1.42	1.41	1.26	1.33	1.38	1.46	1.46	1.09	1.23	1.26	1.33	1.34
Suining	1.97	2.08	2.07	2.23	2.10	1.74	1.75	1.80	1.93	1.93	1.61	1.58	1.63	1.76	1.72	1.88	1.91	1.97	2.17	2.19
Neijiang	2.17	2.13	2.28	2.43	2.59	1.90	1.82	1.84	2.00	2.13	1.98	1.94	1.89	2.07	2.05	2.16	2.07	2.05	2.23	2.45
Leshan	1.18	1.21	1.19	1.26	1.31	1.18	1.17	1.17	1.20	1.18	1.21	1.20	1.22	1.27	1.30	1.38	1.36	1.33	1.33	1.28
Nanchong	1.54	1.49	1.41	1.53	1.57	1.34	1.34	1.32	1.41	1.44	1.69	1.70	1.68	1.79	1.76	1.07	1.09	1.06	1.12	1.14
Meishan	1.88	1.88	1.87	1.87	1.85	1.84	1.86	1.85	1.89	1.91	1.83	1.85	1.89	1.92	1.96	2.05	2.06	2.02	2.00	2.01
Yibin	1.44	1.44	1.47	1.46	1.53	1.46	1.49	1.54	1.60	1.61	1.28	1.35	1.40	1.48	1.43	1.70	1.67	1.72	1.78	1.79
Guang’an	1.73	1.73	1.64	1.61	1.48	1.86	1.90	1.90	1.93	1.89	1.82	1.84	1.80	1.89	1.80	1.94	1.93	1.95	1.98	1.93
Dazhou	1.63	1.71	1.77	1.96	1.99	1.52	1.45	1.46	1.60	1.72	1.64	1.57	1.55	1.68	2.00	1.53	1.47	1.52	1.70	1.81
Ya’an	0.58	0.57	0.55	0.56	0.54	0.88	0.94	0.94	0.92	0.90	0.94	0.99	1.04	1.05	1.08	0.72	0.75	0.72	0.71	0.69
Bazhong	1.55	1.57	1.57	1.63	1.69	1.48	1.50	1.53	1.57	1.52	2.03	1.83	1.86	1.90	1.79	1.15	1.18	1.22	1.23	1.22
Ziyang	2.63	2.58	2.66	2.79	2.88	2.21	2.21	2.24	2.41	2.45	2.19	2.20	2.20	2.32	2.28	2.05	2.22	2.24	2.44	2.53
Aba	0.19	0.17	0.17	0.19	0.18	0.43	0.45	0.44	0.47	0.44	0.30	0.29	0.33	0.37	0.38	0.35	0.32	0.32	0.36	0.33
Ganzi	0.17	0.16	0.15	0.15	0.16	0.36	0.34	0.34	0.37	0.37	0.16	0.16	0.20	0.25	0.27	0.27	0.25	0.25	0.29	0.27
Liangshan	0.63	0.58	0.56	0.56	0.56	0.80	0.80	0.83	0.83	0.87	0.65	0.64	0.67	0.69	0.75	0.78	0.74	0.74	0.75	0.75

### Health resource allocation efficiency values and relaxation in township health centers in Sichuan Province

The overall efficiency, technical efficiency and scale efficiency of township health centers in Sichuan Province in 2017 and 2021 were 1, the returns to scale were constant, and the relaxation was 0, and the DEA was relatively effective. The overall efficiency of township health centers in Sichuan Province in 2018 and 2019 was not 1, the returns to scale were decreasing, and the DEA was relatively ineffective, indicating that the health resource allocation structure was unreasonable. The overall efficiency, technical efficiency, and scale efficiency of township health centers in Sichuan Province in 2020 were not 1, indicating that the problem of insufficient input and inefficient health resources allocation existed simultaneously (Table 3).

**Table 3:** Health resource allocation efficiency values and relaxation in township health centers in Sichuan Province.

Year	Overall efficiency	Technical efficiency	Scale efficiency	Returns to scale	Beds	Health technicians	Licensed (assistant) physicians	Registered nurses	The number of consultations	Hospital admissions	Relatively efficiency status
2017	1.000	1.000	1.000	Constant	0.000	0.000	0.000	0.000	0.000	0.000	Efficient
2018	0.991	1.000	0.991	Decreasing	409.811	1 221.272	0.000	1 379.053	0.000	398 475.775	Inefficient
2019	0.985	1.000	0.985	Decreasing	0.000	444.242	0.000	2 728.826	0.000	120 688.484	Inefficient
2020	0.911	0.916	0.994	Decreasing	0.000	600.298	147.625	3 575.194	0.000	277 469.225	Inefficient
2021	1.000	1.000	1.0000	Constant	0.000	0.000	0.000	0.000	0.000	0.000	Efficient

### Health resource allocation efficiency values of township health centers in each region of Sichuan Province

The efficiency of health resource allocation in township health centers in each region of Sichuan Province is shown in Table 4. The overall efficiency, technical efficiency and scale efficiency of all health resources in Mianyang and Ziyang were 1, and the DEA was relatively effective. The overall efficiency, technical efficiency and scale efficiency of all health resources in Suining, Neijiang, Yibin, Aba and Ganzi were not 1, and the DEA was relatively ineffective, indicating that the problem of insufficient input and inefficient allocation of health resources existed simultaneously.

**Table 4:** Health resource allocation efficiency values of township health centers in each region of Sichuan Province.

Region	2017			2018			2019			2020			2021		
	Overall efficiency	Technical efficiency	Scale efficiency	Overall efficiency	Technical efficiency	Scale efficiency	Overall efficiency	Technical efficiency	Scale efficiency	Overall efficiency	Technical efficiency	Scale efficiency	Overall efficiency	Technical efficiency	Scale efficiency
Chengdu	0.859	1.000	0.859	0.861	1.000	0.861	0.933	1.000	0.933	0.917	1.000	0.917	0.975	1.000	0.975
Zigong	0.992	0.992	1.000	1.000	1.000	1.000	1.000	1.000	1.000	0.951	0.962	0.989	0.939	0.942	0.997
Panzhihua	1.000	1.000	1.000	0.920	1.000	0.920	0.929	1.000	0.929	1.000	1.000	1.000	1.000	1.000	1.000
Luzhou	0.963	0.973	0.990	0.921	0.960	0.958	0.985	1.000	0.985	0.898	0.899	0.999	0.930	0.947	0.982
Deyang	1.000	1.000	1.000	1.000	1.000	1.000	1.000	1.000	1.000	0.934	0.935	0.999	1.000	1.000	1.000
Mianyang	1.000	1.000	1.000	1.000	1.000	1.000	1.000	1.000	1.000	1.000	1.000	1.000	1.000	1.000	1.000
Guangyuan	0.825	0.828	0.996	0.811	0.814	0.997	0.794	0.801	0.991	0.856	0.861	0.994	1.000	1.000	1.000
Suining	0.775	0.841	0.921	0.827	0.901	0.917	0.778	0.864	0.900	0.698	0.803	0.869	0.652	0.741	0.880
Neijiang	0.955	0.973	0.981	0.860	0.889	0.967	0.914	0.939	0.974	0.812	0.884	0.919	0.820	0.857	0.957
Leshan	0.960	0.961	1.000	0.932	0.932	1.000	0.953	0.957	0.997	0.947	0.949	0.997	0.950	0.952	0.998
Nanchong	1.000	1.000	1.000	1.000	1.000	1.000	1.000	1.000	1.000	1.000	1.000	1.000	0.983	1.000	0.983
Meishan	0.947	0.954	0.993	0.945	0.952	0.992	1.000	1.000	1.000	0.904	0.915	0.988	0.872	0.880	0.990
Yibin	0.782	0.795	0.983	0.780	0.784	0.995	0.826	0.830	0.996	0.848	0.849	0.998	0.894	0.898	0.996
Guang’an	0.859	0.861	0.997	0.794	0.801	0.991	0.850	0.852	0.997	0.937	0.937	1.000	1.000	1.000	1.000
Dazhou	1.000	1.000	1.000	0.890	1.000	0.890	1.000	1.000	1.000	1.000	1.000	1.000	0.930	1.000	0.930
Ya’an	0.856	0.864	0.991	1.000	1.000	1.000	1.000	1.000	1.000	1.000	1.000	1.000	1.000	1.000	1.000
Bazhong	0.895	0.918	0.974	0.915	0.962	0.951	0.918	0.960	0.957	0.917	0.992	0.924	1.000	1.000	1.000
Ziyang	1.000	1.000	1.000	1.000	1.000	1.000	1.000	1.000	1.000	1.000	1.000	1.000	1.000	1.000	1.000
Aba	0.538	0.668	0.806	0.461	0.704	0.655	0.433	0.718	0.602	0.501	0.784	0.638	0.514	0.882	0.583
Ganzi	0.784	0.928	0.845	0.755	0.969	0.779	0.617	0.794	0.777	0.589	0.670	0.880	0.603	0.705	0.854
Liangshan	1.000	1.000	1.000	0.725	0.735	0.986	0.730	0.741	0.986	0.808	0.817	0.988	0.719	0.736	0.977

## Discussion

The health resources of township health centers in Sichuan Province have been developed rapidly, which indicates that the standardization construction of township health centers in Sichuan Province has achieved certain results, and township health centers have played an active role in the primary medical and health care system. Influenced by COVID-19, which began in December 2019, health resources in township health centers in Sichuan Province grew slightly in 2020–2021, but health service utilization trended downward, with the number of consultations dropping by more than 3.18 million and hospital admissions dropping by more than 140,000. In response to COVID-19, Sichuan Province has mainly drawn medical and nursing staff from various hospitals, which has had less impact on the equity of health resources.COVID-19 has had a greater impact on the utilization of health services, and although some patients have chosen to receive online healthcare services, the number of consultations and hospital admissions have still declined significantly. Therefore, it is important to emphasize the rational deployment of health resources and the stockpiling of emergency supplies in response to public health emergencies, and to enhance the public health service capacity of township health centers.

The Gini coefficient of health resource allocation by population in township health centers in Sichuan Province is below 0.3, while the Gini coefficient by geography is above 0.6, which indicates that the equity of health resource allocation by population in township health centers in Sichuan Province is better than that by geography. We believe there are two reasons for this. On the one hand, health planning usually uses the number of health resources per 1,000 population as the allocation standard, thus paying less attention to the impact of geographical area on health resource allocation. The long-standing urban-rural dual structure, the overall health resource allocation is toward cities and towns [17], resulting in a large gap of health resources in rural areas. With the development of social economy, a large number of people are concentrated in urban areas, which makes the health resources in rural areas grow slowly, making it difficult to meet the health care needs of rural residents. Especially in the sparsely populated and economically underdeveloped minority areas, the lack of health resources is more serious. On the other hand, urban areas have large populations, more developed socioeconomics, convenient transportation, and more active healthcare markets. However, the opposite is true in rural areas, where the health care awareness of rural residents is relatively low and the utilization of medical and health services is relatively low, resulting in insufficient development of health resources and insufficient medical service capacity in rural areas [18]. The inequality of health resources allocation by population is gradually increasing, which further indicates that the urban population concentration has led to the deterioration of health resources allocation in township health centers [19], and the construction of township health centers in remote and poor areas is more difficult. The Gini coefficient of health resources allocation by geography in township health centers is more than 0.6, and is in a highly inequitable and dangerous state, which also prompts us to pay great attention to the health resources allocation in remote and poor areas [20]. Therefore, it is necessary to strengthen the construction of the medical and health service system of township health centers and to make reasonable planning for the allocation of health resources by integrating population and geographical factors. Strengthen the health human resources of township health centers in remote and poor areas through a variety of ways [21], such as counterpart support from resource-rich areas to weak areas and targeted training of medical students.

Integrating population and geographic factors, the Ws in Panzhihua, Ganzi, Aba and Liangshan is less than 1, indicating that the allocation of health resources in these regions is lower than the average level in Sichuan Province. We believe there are three reasons for this. First, Ganzi, Aba and Liangshan are regions where ethnic minorities are concentrated. Due to the weak historical foundation, insufficient financial input and late development of health care, the problem of shortage and inequality of health resources in these regions has not yet been solved. Second, Ganzi, Aba, Liangshan and other regions have poor socio-economic development, financial investment in medical and health care is insufficient, resulting in the slow development of health resources in township health centers. These regions have lower salaries for medical professionals, the introduction and retention of medical professionals in township health centers is insufficient, and it is also easy to lose them to regions with higher economic levels [22], resulting in a shortage of medical professionals in township health centers [23]. These regions also have conditions of relatively inaccessible social transportation, high cost of medical services, and the limited financial input from the administration makes it difficult to cover all regions. Third, the residents of minority regions in Sichuan Province are most interested in the convenience of services when choosing primary health care services, and it is difficult for township health centers to cover all residents. These regions are vast and sparsely populated, so rural residents who tend to choose medical institutions that are closer or higher-level medical institutions [24], fewer people visit local township health centers, resulting in a lack of motivation for health service development. Therefore, it is necessary to develop the social economy based on regional advantages, further strengthen the construction of township health centers in Panzhihua, Ganzi, Aba and Liangshan, and build village health rooms in places where township health centers are insufficiently covered [25], so as to expand the coverage of the primary health care system and meet the people’s demand for medical care. At the same time, it should strengthen the support and assistance from resource-rich areas to weak areas in order to improve the medical service capacity of township health centers, so as to retain local residents to visit local doctors.

The overall efficiency of township health centers in Sichuan province is not 1 in 2018–2020, and the DEA is relatively inefficient in all regions except Nanchong and Ziyang in 2017–2021, which indicates that the health resource allocation efficiency of township health centers in Sichuan province is low, and the problems of insufficient input and inefficient health resource allocation exist simultaneously. We believe there are three reasons for this. First, the supply scale of medical and health services in township health centers is still insufficient, and township health centers still need to increase financial input. In recent years, the infrastructure and equipment of township health centers have been improved, but the training of health personnel is still insufficient, and the capacity of health and medical services is still relatively low. It cannot comprehensively improve the efficiency of medical services simply by relying on measures such as increasing medical equipment and expanding the scale of services. Second, with the change of disease spectrum and the development of medical technology, the inadequate level of medical technology in township health centers has hindered the utilization of medical services by local residents [26]. The lack of promotion of new technologies and programs in township health centers, and the level of treatment technology make it difficult to retain local people who seek medical care, resulting in the underutilization of health resources. The relatively low capacity of medical and technical services in township health centers, combined with the fact that public health services take up a large amount of working time, leads to the underprovision and underutilization of basic medical and health services [27]. Thirdly, factors such as weak market awareness, poor management level and undeveloped management concept of township health center managers have affected the improvement of the efficiency of township health center services. Township health centers are tired of meeting their daily work, with fewer opportunities for further training and study, and the managers have a low level of professionalism, making it difficult to cope with the complex and changing medical market [28]. Therefore, according to the socio-economic conditions and the demand for medical services in the region, we should improve the construction of medical human resources, promote the growth of facilities and equipment and medical and technical service capacity, and enhance the efficiency of medical service utilization [29]. We will make full use of the graded diagnosis and treatment system to guide the development of medical technology in township health centers, to guide the downward flow of high-quality health resources and cross-regional flow, to shift from rough management to refined management in the management style, and to improve the efficiency of medical and health services and management efficiency [30].

## Limitations

Although we have used the Lorenz curve, Gini coefficient, HRDI, and DEA to evaluate the equity and efficiency of health resource allocation in township health centers in Sichuan Province, there are still some limitations to this study. First, the evaluation indicators were selected based on previous relevant studies, which are commonly used in similar studies. Second, although we evaluated the equity and efficiency of health resource allocation in township health centers in Sichuan Province, but the factors influencing health resource allocation are various, this study did not consider the actual health status and health service demand in different regions, and did not consider the quality of health resources. Third, the construction of township health centers is more dependent on financial input, and this study does not analyze the financial input of township health centers.

## Conclusions

To improve the equity and efficiency of health resource allocation in township health centers is important to promote the development of primary health care system in Sichuan Province. The main findings and recommendations of this study are as follows. From 2017 to 2021, the Gini coefficient of health resources by population in township health centers in Sichuan Province is below 0.3, and the Gini coefficient of health resources by geography is above 0.6, and the equity of health resources allocation by population in township health centers in Sichuan Province is better than that by geography. Integrating the factors of population and geographic area, the Ws of health resources in Panzhihua, Ganzi, Aba, and Liangshan are less than 1, indicating that the allocation of health resource in these regions is lower than the average level in Sichuan Province. The overall efficiency of township health centers in Sichuan Province is not 1 in 2018–2020, and the DEA is relatively ineffective in all regions except Mianyang and Ziyang in 2017–2021, and the health resource allocation efficiency of township health centers in Sichuan Province is low. The administrative department should strengthen the construction of medical and health service systems in township health centers, and make reasonable planning for the allocation of health resources in township health centers by integrating population and geographical factors, especially to strengthen the allocation of health resources in weak regions.

## Supporting information

S1 Table Data on health resources and service utilization in township health centers in 21 cities and states in Sichuan Province, 2017–2021. (XLSX)
